# Development and application of a home-based exercise program for patients with cardiovascular disease: a feasibility study

**DOI:** 10.1186/s13102-024-00835-3

**Published:** 2024-02-21

**Authors:** Mi Kyung Lee, Chan Joo Lee, Seon Young Goo, Tae Ho Lee, Jin Young Moon, Jiyoung Jung, Min Jung Kim, Sang Hee Shin, Jong Nam Kim, Sung Nim Han, Jung Eun Lee, Jong Young Lee, Ick-Mo Chung, Justin Y. Jeon

**Affiliations:** 1 Frontier Research Institute of Convergence Sports Science, FRICSS, Yonsei University, Seoul, South Korea; 2 Division of Cardiology, Department of Internal Medicine, Severance Hospital, Yonsei University College of Medicine, Seoul, South Korea; 3 Department of Sport Industry Studies, Yonsei University, Seoul, South Korea; 4 Cardiology Division, Ewha Womans University Mokdong Hospital, Seoul, South Korea; 5 Department of Educational Psychology, Seoul Women’s University, Seoul, South Korea; 6 Department of Food and Nutrition, College of Human Ecology, Seoul National University, Seoul, South Korea; 7 Division of Cardiology, Kangbuk Samsung Hospital, Sungkyunkwan University School of Medicine, Seoul, South Korea; 8 Division of Cardiology, Department of Internal Medicine, Mokdong Hospital, School of Medicine, Ewha Womans University, Seoul, South Korea; 9 Division of Cardiology, Ewha Womans University Mokdong Hospital, 1071 Anyangcheon-ro, Yangcheon-gu, Seoul, Republic of Korea; 10 Exercise Medicine Center for Diabetes and Cancer Patients, ICONS, Yonsei University, Seoul, South Korea; 11 Cancer Prevention Center, Yonsei Cancer Center, Yonsei University College of Medicine, Seoul, Republic of Korea; 12 Department of Sport Industry Studies and Exercise Medicine Center for Diabetes and Cancer Patients, Yonsei University, 50 Yonsei-ro, Seodaemun-gu, Seoul, Republic of Korea

**Keywords:** Home-based cardiac rehabilitation, Physical activity, Home-based exercise program, Cardiovascular disease

## Abstract

**Background:**

Cardiac rehabilitation (CR) is recommended for patients with cardiovascular disease. However, the participation and completion rates for hospital-based CR are low, and home-based CR has been suggested as an alternative. This study aimed to develop a home-based CR program and assess the feasibility of the program over a 6-week period in patients with left ventricular dysfunction or a history of myocardial infarction.

**Methods:**

This feasibility study consisted of two phases. The initial phase (Study 1) focused on developing the home-based exercise protocol. Systematic approaches to developing evidence-based home-based exercise intervention were implemented including systematic review, patient surveys, and expert consensus. Study 2 aimed to evaluate the feasibility of a 6-week home-based CR program that was based on the results of Study 1. Study 2 included two exercise education sessions and four telephone counseling sessions. During this stage of the exercise program, the participants exercised on two separate days and their experiences while performing the aerobic and resistance exercises were surveyed. Eight participants participated in Study 1 and 16 participated in Study 2.

**Results:**

Participants expressed overall satisfaction with the exercise program in Study 1. Heart rate increased in response to exercise, but this did not correspond with perceived exertion. The aim of the home-based CR exercise program was for participants to achieve exercise goals (≥150 min/week of aerobic type exercises as well as at least twice weekly resistance exercise using own body weights). We aimed to increase compliance and adherence to the home-based CR program. In Study 2, 13 out of 16 participants (81.3%) completed the 6-week home-based CR program, with a participation rate of 100% in both exercise education and phone counseling sessions. Adherence to the home-based exercise protocol was 83.1% and no serious adverse events were observed. At the beginning of the study, only three out of 13 participants (23.1%) met the requirements for both aerobic and resistance exercises, but at the end of the 6-week program, 10 out of 13 participants (76.9%) fulfilled the requirements.

**Conclusion:**

The exercise program developed in this study was safe and feasible, and the 6-week home-based CR program was feasible for patients with cardiovascular disease without any reported adverse effects.

**Supplementary Information:**

The online version contains supplementary material available at 10.1186/s13102-024-00835-3.

## Background

Cardiovascular disease (CVD) is the top leading cause of death and disease burden globally. More than 19 million deaths were attributed to CVD globally, which amounted to an increase of 18.7% from 2010 [1]. In Korea, CVD is the second highest cause of death; moreover, deaths due to ischemic heart disease, especially myocardial infarction (MI), and heart failure are on the rise [2]. As the number of patients with CVD increases, the secondary prevention of CVD has become a critical issue.

Cardiac rehabilitation (CR) is an evidence-based intervention that uses exercise training, health behavior modification, and patient education to improve secondary prevention outcomes in patients with CVD [3]. Evidence supports the safety and effectiveness of CR [4, 5], and national guidelines recommend CR in CVD patients. The CR program has been given a Class I recommendation from the American College of Cardiology, and the European Society of Cardiology, which identified exercise therapy as a central element [6–8]. Anderson et al. systematically reviewed 63 studies involving 14,486 participants with a median follow-up of 12 months and reported that CR reduced cardiovascular mortality by 26.9% and the incidence of hospital readmissions by 18% [9]. Another recent systematic review that included seven studies (581 patients) with a median of 12-month follow-up reported that exercise-based CR reduced acute MI recurrence by 67% and hospital readmission by 86% in patients with stable angina [10].

Although the efficacy and safety of CR have been firmly demonstrated, it has been underutilized globally [11–13]; moreover, the underutilization of CR became more prevalent during the COVID-19 pandemic [14]. In South Korea, only 1.5% of acute MI cases undergo hospital-based CR [15]. Barriers to participating in hospital-based CR include distance from the hospital, cost, transportation problems, family responsibilities, lack of knowledge about CR, and time constraints [16]. Home-based CR has been suggested as an alternative to overcome these barriers and increase patients’ level of participation in CR. A recent systematic review conducted by Taylor et al. [17] reported that home- and center-based CR were similar in terms of mortality, exercise capacity, and health-related quality of life, except for higher levels of trial completion in the home-based group. Therefore, the AACVPR/AHA/ACC released a scientific statement on home-based CR emphasizing its importance, especially for clinically stable low- to moderate-risk patients who are eligible for CR but cannot attend a traditional center-based CR program [3]. However, home-based CR programs vary significantly across studies, and studies often do not report the detailed process of home-based CR programs [18].

The relationship between patients and health professionals, barriers, and facilitators of exercise vary significantly depending on culture and country; therefore, it is important to develop a tailored, scientifically evidence-based, home-based CR program according to participants’ characteristics and cultural background. However, there is no study which report process of home-based CR program in Korea, where a hospital-based CR is underutilized. In this study, we describe the process of developing a home-based exercise program (study 1) and report the feasibility and safety of implementing a home-based CR program in an outpatient setting (Study 2).

## Methods

### Study design and procedure

For the study 1, a home-based exercise program was developed through a systematic process outlined by An et al. [19]. The process included a systematic literature review to understand hospital- and home-based CR; a survey of 189 patients with CVD to assess their physical activity (PA) participation levels, attitudes, barriers, and facilitators of exercise PA, and the formation of an expert committee composed of three cardiologists and two exercise specialists to establish goals, implementation strategies, and precautions for home-based CR.

Based on an expert committee meeting, preliminary exercise programs were developed with the goal of helping patients with CVD to follow exercise guidelines for both aerobic and resistance exercises. Because physical fitness and exercise experience varied among patients, walking was recommended as the main type of aerobic exercise, and an aerobic exercise video was produced for patients when outdoor walking was not feasible. Considering that participation in resistance exercise at a fitness center may not be feasible for many patients with CVD, a calisthenic exercise program was developed. The program consisted of eight resistance exercises that utilized the participant’s own body weight. The number of repetitions, sets, and rest intervals was determined based on the initial fitness levels and joint conditions of the participants (Supplementary Table 1, Supplementary Material 1 and 2).

The study 2 was then conducted to assess the feasibility of following the exercise video and to measure heart rate responses and the rate of perceived exertion (RPE) during exercise (Study 1). During this stage of development of the exercise program, the participants exercised on two separate days and their experiences while performing the aerobic and resistance exercises were surveyed (Supplementary Table 2). Subsequently, an expert panel consisting of three cardiologists, two exercise specialists, two clinical psychologists, and two clinical nutritionists reviewed and revised the home-based CR program based on heart rate data, RPE response, and survey data. A feasibility study was then conducted to examine the implementation of a home-based exercise program in conjunction with dietary and psychological counseling over a 6-week period in a tertiary hospital setting (Study 2).

Data from the Study 1 and feasibility studies (Study 2) are reported and discussed. It is worth noting that the implementation of home-based CR includes psychological and nutritional counseling in addition to home-based exercise rehabilitation. This study was approved by the Institutional Review Board of Yonsei University College of Medicine (approval Nos: 4-2021-0576 and 4-2021-0935).

### Participants

**Fig. 1:**
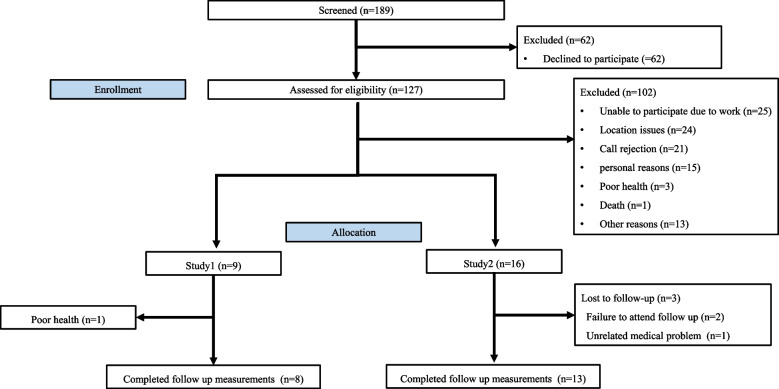
CONSORT Diagram

From September 2021 to July 2022, we recruited participants from Yonsei Severance Hospital in Seoul, Republic of Korea. Patients with heart failure with reduced ejection fraction (HFrEF) or MI were included in the study. The study details were explained to 127 patients who met the inclusion criteria during their visit to the Cardiology Clinic at Severance Hospital. Nine participants (MI 2, MI with HFrEF 3, and HFrEF 4) were enrolled in Study 1, and 16 participants (MI 3, MI with HFrEF 6, and HFrEF 7) were enrolled in Study 2 (Fig. 1, Table 1). All participants who agreed to participate in the study provided written informed consent.

**Table 1:** Participant characteristics

	Study 1	Study 2
	(*n* = 8)	(*n* = 13)
Sex		
Male	6 (75.0)	10 (76.9)
Female	2 (25.0)	3 (23.1)
Age (years)	56.4 ± 10.6	60.9 ± 8.1
BMI (kg/m^2^)	27.3 ± 4.8	26.2 ± 4.0
Education		
High School	5 (62.5)	5 (38.5)
College or higher	3 (37.5)	8 (61.5)
Marital status		
Married	6 (75.0)	10 (76.9)
Never been married	1 (12.5)	0
Divorced	1 (12.5)	3 (23.1)
Employment		
Employed	2 (25.0)	7 (53.8)
Not employed	6 (75.0)	6 (46.2)
Monthly income (10,000 won)		
< 300	4 (50.0)	5 (38.5)
300 ~ 499	2 (25.0)	6 (46.2)
≥ 500	2 (25.0)	2 (15.4)
Drinking		
Yes	3 (37.5)	4 (30.8)
No	5 (62.5)	9 (69.2)
Smoking		
Yes	1 (12.5)	1 (7.7)
No	7 (87.5)	12 (92.3)
Hypertension	5 (62.5)	10 (76.9)
Diabetes	4 (50.0)	7 (53.8)
Hyperlipidemia	4 (50.0)	6 (50.0)
Chronic kidney disease	1 (12.5)	3 (25.0)
Heart failure	6 (75.0)	10 (76.9)
Myocardial infarction	5 (62.5)	6 (46.2)

Data are presented as mean ± standard deviation or number (%). *BMI* body mass index

### Study design and outcome measure

Study 1 involved a 2-day exercise session, in which participants completed separate sessions of aerobic and resistance exercises each day. All exercise programs were conducted under the supervision of exercise specialists at the Yonsei University Fitness Center. On day 1, the participants engaged in three different types of aerobic exercises, each lasting approximately 15 min with three different levels of intensity (low, moderate, and high). Participants’ heart rates and RPE were monitored during the exercises. On day 2, the participants completed two sets of eight different calisthenic exercises, and their heart rates and RPE were monitored. If the exercise became too challenging or difficult to follow, participants were instructed to take a rest. Additionally, the participants were asked to complete surveys to assess their level of satisfaction with each exercise type (Supplementary Table 2).

In Study 2, upon enrollment, the participants’ levels of PA and physical functions were assessed. Two exercise education sessions were conducted in the 2nd and 4th week, along with four phone counseling sessions in the 1st, 3rd, 5th, and 6th week. The aim of the home-based CR exercise program was to increase the participants’ moderate-intensity exercise to 150 min or more per week, with resistance exercise utilizing their body weight at least twice a week. The intensity of aerobic exercise was set at 40–80% of heart rate reserve or 11–16 on the RPE scale. The participants received education from exercise specialists to perform the prescribed calisthenic exercises correctly, were encouraged to comply with the exercise program, and were monitored weekly. They were provided with an exercise diary and videos containing stretching, calisthenics, and aerobic exercises (walking and videos), which could be performed daily at home.

To assess the feasibility of the home-based exercise program, the participants’ compliance with the exercise sessions was monitored by conducting two face-to-face sessions and four telephone counseling sessions. The participants were also asked to write in an exercise diary to record their participation in the prescribed exercises. These measures were adopted to ensure that the participants were able to follow the prescribed exercise program (acceptability and fidelity) and to identify any issues that may have risen during the intervention. Additionally, the participants were asked to complete surveys to assess their level of satisfaction with each component of a home-based CR program (Supplementary Table 3).

PA levels were evaluated at baseline and at the end of the 6th week. The proportion of participants who met the PA guidelines for both aerobic and resistance exercises was also compared at baseline and at the 6th week of the intervention. PA was assessed using the Global Physical Activity Questionnaire (GPAQ). Three tests were performed to measure physical function: hand grip strength, sit-to-stand, and 6-minute walk tests. Handgrip strength was measured of both hands using a Takei A5401 hand grip dynamometer (Takei Scientific Instruments, Niigata, Japan). For the sit-to-stand test, the participants were asked to complete as many full stands as possible within 30 s. For the 6-minute walk test, participants were asked to walk for a total of 6 min and cover as much distance as possible.

### Statistical analysis

Descriptive statistics were used to analyse the results of our studies. Continuous variables are expressed as means and standard deviations and where data was not normally distributed, median, and interquartile ranges were reported. Categorical variables are presented as percentages. and chi-squared tests were used to detect differences between the groups. The statistical software SPSS version 25.0 (IBM Corp, Armonk, NY, USA).

## Results

### Study 1

In Study 1, the participants’ heart rates increased during low-, moderate-, and high-intensity aerobic exercise sessions, reaching a maximum of 122 bpm (range: 75–122 bpm), 124 bpm (range: 82–124 bpm), and 129 bpm (range: 81–129 bpm), respectively. Six of the eight participants completed all three exercise sessions at different intensities. One participant’s heart rate exceeded 100 bpm during low-intensity exercise, the heart rates of two participants exceeded 100 bpm during moderate-intensity exercise, and all six participants’ heart rates exceeded 100 bpm during high-intensity exercise. In the resistance exercise sessions, during the first set, heart rates reached a maximum of 150 bpm (range: 88–150 bpm), and during the second set, heart rates reached a maximum of 108 bpm (range: 93–108 bpm). Four out of eight participants completed both sets of exercise. The RPE increased during low-, moderate-, and high-intensity aerobic exercises, as well as resistance exercises, reaching maxima of 15, 15, 17, and 17, respectively (Tables 2 and 3). The exercise program consisting of aerobic and resistance exercises received high satisfaction ratings from participants, with an average score of 4.3 out of 5.0 points. Participants also perceived the program as beneficial for their treatment.

**Table 2:** Changes in HR and RPE during aerobic exercise (Study 1; *n* = 8)

ID	Age	Sex	HRR 40 ~ 80%	Low intensity aerobic exercise	Moderate-intensity aerobic exercise	High-intensity aerobic exercise
2	73	M	99–130	HR^a^: 70–82–99-82	HR: 67–90–94-93	HR: 77–73–73-81
				RPE^b^: 11–15	RPE: 11–15	RPE: 11–13
3	54	M	102 ~ 145	HR: 59–72–75-75	HR: 59–82–81-82	
				RPE: 7–15	RPE: 11–15	
4	53	F	105 ~ 146	HR: 73–75–77-88	HR: 68–97–100-105	HR:71–92–96-109
				RPE: 9–13	RPE: 9–15	RPE: 11–13
5	51	M	105 ~ 147	HR: 64–75–75-82	HR: 68–86–84-87	HR:69–97–97-102
				RPE: 11–13	RPE: 11–15	RPE: 13–17
6	45	M	106 ~ 151	HR: 61–74–81-85	HR: 61–86–91-90	
				RPE: 12–13	RPE: 12–14	
7	44	M	116 ~ 156	HR: 79–112–116-122	HR: 83–118–124-124	HR: 88–123–127-129
				RPE: 7–11	RPE: 7–13	RPE: 9–15
8	68	F	103 ~ 135	HR: 76–84–83-84	HR: 79–88–93-93	HR:77–96–98-102
				RPE: 7–9	RPE: 7–11	RPE: 7–13
9	63	M	106 ~ 140	HR: 80–93–94-95	HR: 79–100–107-104	HR: 81–104–107-110
				RPE: 7–13	RPE: 9–11	RPE: 11–13

^a^HR: changes in HR during exercise, resting HR– HR after 1 set of exercise – HR after 2 sets of exercise – HR after 3 sets of exercise, ^b^RPE (Rate of Perceived Exertion): changes in RPE, resting RPE – RPE after 3 sets of exercise

**Table 3:** Changes in HR and RPE during resistance exercise (Study 1; *n* = 7)

ID	Age	Sex	HRR 40 ~ 80%	1 set	2 set
2	73	M	99–130	HR^a^: 66–82	HR: 71–85
				Peak HR: 100	Peak HR:93
				RPE^b^: 11–15	RPE: 11–15
4	53	F	105 ~ 146	HR: 65–90	
				Peak HR: 91	
				RPE: 11–15	
5	51	M	105 ~ 147	HR: 72–84	HR: 80–85
				Peak HR:90	Peak HR:94
				RPE: 11–12	RPE: 11–15
6	45	M	106 ~ 151	HR: 120–125	
				Peak HR:150	
				RPE: 15–16	
7	44	M	116 ~ 156	HR: 80–101	HR:75–107
				Peak HR:101	Peak HR:107
				RPE: 7–17	RPE: 9–15
8	68	F	103 ~ 135	HR: 79–88	
				Peak HR:88	
				RPE: 7–12	
9	63	M	106 ~ 140	HR: 84–105	HR:85–108
				Peak HR:105	Peak HR:108
				RPE: 8–13	RPE: 11–15

^a^HR: changes in HR, HR before exercise– HR after exercise, ^b^RPE (Rate of Perceived Exertion): changes in RPE, RPE before exercise – RPE after exercise

From study 1, a home-based CR program was developed to be implemented in Study 2. Table 4 showed detailed program.

**Table 4:** Home-based cardiac rehabilitation program for 6 weeks

Exercise Goals	1. At least 150 min of moderate to vigorous aerobic exercise 2. 1–3 sets of Calisthenics (10 repetitions of 8 exercise)	40–80% of HRR No restriction on rest interval
Intervention Strategies	1. Face-to-Face Exercise education sessions (1st and 3rd week of intervention) 2. Weekly Phone Follow up 3. Providence of Smart watch to monitor physical activity and heart rate during exercise 4. Providence of Exercise Diary	Face-to-Face education session covers proper walking and exercise technique, usage of smart watch and exercise diary
Exercise Diary	Exercise diary covers goal setting, strategies to achieve goals, exercise video QR link, explanation on the benefit of exercise for cardiac patients, detailed exercise instruction with pictures. Guidance of exercise modification strategies when patients have joint pain. Exercise recommendation when patients have comorbidities such as diabetes, kidney disease, osteoarthritis etc.	

*HRR* Heart rate reserve

### Study 2

In Study 2, 13 out of 16 participants (81.3%) completed a 6-week home-based CR program. Loss to follow-up after the baseline assessment was identified as the reason for dropouts. The participation rate in the exercise education and phone counseling sessions was 100%, and adherence to the exercise protocol (moderate-intensity aerobic exercise for more than 150 min and resistance exercise for more than 2 days per every week) was 83.1%. No serious adverse events were observed.

Before the intervention, eight (61.5%) participants were engaged in moderate-to vigorous intensity aerobic exercise for more than 150 min per week, and 6 weeks later, 11 (84.6%) participants achieved this aerobic exercise goal. Moreover, prior to the intervention, only three (23.1%) participants participated in both moderate- and vigorous intensity aerobic exercise for more than 150 min per week and resistance exercise for more than twice a week. However, at the end of the Study 2, 10 (76.9%) out of 13 participants met both aerobic and resistance exercise goals (Table 5).

**Table 5:** Changes in meeting the physical activity goal in the Study 2

Variables	Total (*n* = 13)	
	baseline	6 weeks
≥150 minutes exercise per week^a^	8 (61.5)	11 (84.6)
≥150 minutes exercise^a^ and resistance exercise^b^ per week	3 (23.1)	10 (76.9)

Data are presented as number (%), ^a^Moderate to vigorous-intensity exercise = (minutes of vigorous-intensity exercise per week × 2) + (minutes of moderate-intensity exercise per week), ^b^2 days of resistance exercise

Vigorous intensity exercise levels increased from 0 (0–180) min/week to 60(0–300) min/week and moderate intensity exercise levels increased from 120(0–210) min/week to 240(120–355) min/week after intervention. The frequency of resistance exercise increased from 0(0–1.5) day/week to 2 (2–5) day/week. No change in the physical fitness were observed (Table 6).

**Table 6:** Changes in physical activity level and fitness in the Study 2

Variables	Total (*n* = 13)	
	baseline	6 weeks
Physical activity		
Vigorous-intensity exercise (min/week)	0 (0–180)	60 (0–300)
Moderate-intensity exercise (min/week)	120 (0–210)	240 (120–355)
Transport activity (min/week)	135 (30–240)	180 (105–300)
Vigorous-intensity work (min/week)	0	0 (0–20)
Moderate-intensity work (min/week)	40 (5–220)	30 (20–60)
Total walking (min/week)	240(145–390)	420 (180–555)
Sedentary behavior (min/day)	480 (300–720)	480 (210–720)
Strength exercise (day/week)	0 (0–1.5)	2 (2–5)
Physical fitness		
Right hand grip strength (kg)	36.4 (23.9–42.5)	36.0 (26.4–43.7)
Left hand grip strength (kg)	36.3 (23.9–43.2)	36 (26.5–41.3)
Sit-to-stand test (rep/30 sec)	18 (12–28.5)	20 (15.5–26.0)
6-min walk test (m/6 min)	587 (465–613.5)	572 (509–603.5)

Data are presented as median (inter quartile range)

In Study 2, the participants reported high satisfaction with exercise education (4.5/5.0), exercise videos (4.4/5.0), and telephone-based exercise counseling (4.7/5.0). However, some participants reported difficulty in the regular use of the exercise diary, with an average satisfaction score of 3.8/5.0 points (Table 7).

**Table 7:** Satisfaction with home-based exercise program in the Study 2

Variables	Median (IQR)
Satisfaction with exercise education	5 (4–5)
Appropriateness of exercise time and intensity	5 (3.5–5)
Satisfaction with telephone counseling	5(4–5)
Appropriateness of telephone counseling frequency	5(4–5)
Utilization of exercise diary writing	4(3–5)
Utilization of exercise videos	5(4–5)

Data are presented as median (inter quartile range)

## Discussion

In this study, we describe the development of a home-based exercise CR program. In Study 1, eight participants completed aerobic and resistance exercise sessions, during which their heart rates and RPE were monitored. Heart rate data indicated a low-to-moderate exercise intensity, whereas RPE showed a moderate-to-vigorous intensity. As a result, a home-based CR program was successfully developed by study 1 and was used for Study 2. In Study 2, we observed high compliance rates for face-to-face exercise education and home-based exercise sessions, which resulted in an increase in PA participation. Moreover, a higher proportion of the participants met both the aerobic and resistance exercise recommendations after the intervention. Our findings suggest that home-based exercise programs are safe and feasible for outpatient implementation, with high compliance and adherence rates.

The heart rate of the participants in Study 1 did not increase proportionally with their RPE. This phenomenon, known as chronotropic insufficiency, can be attributed to various factors, including the use of antihypertensive medications, such as beta-blockers. As most participants were administered beta-blockers, their heart rate response to exercise was blunted, and they subjectively perceived exercise intensity as vigorous. This observation indicates that monitoring exercise intensity during CR should not rely solely on the heart rate response during exercise. Another possible explanation for the lack of an increase in heart rate during exercise is the participants’ inadequate exercise skills and exercise intolerance, which could elicit insufficient physiological responses to exercise. This finding underscores the importance of exercise education sessions, in which proper exercise techniques can be taught gradually to increase exercise intensity and duration. When participants were unable to perform the exercises correctly, they elicited insufficient physiological responses and increased the risk of injury. Therefore, during the feasibility study (Study 2), we included two face-to-face exercise education sessions (one-on-one supervised exercise sessions with an exercise specialist) during the 6-week intervention program.

A blunted heart rate response during exercise CR is an important issue among patients on beta-blockers [20]. In healthy individuals, exercise can increase heart rate up to approximately 2.5-fold, whereas stroke volume increases only approximately 1-to 1.5-fold; however, these variables may vary according to age, sex, and fitness levels [21]. As a result, the contribution of chronotropic insufficiency to exercise intolerance is likely greater in patients with small and stiff left ventricles. A recent study that pooled data from studies examining the relative contribution of hemodynamic variables to exercise limitations suggested that chronotropic insufficiency was the most relevant contributor to functional impairment [22]. Another recent single-blind, randomized, crossover study showed that withdrawal of beta-blockers resulted in 15 and 30% increases in peak VO_2_ and heart rate, respectively, during cardiopulmonary exercise test [23]. Although identifying the mechanisms underlying the lack of heart rate response during exercise is beyond the scope of our pilot study, it is essential for both exercise therapists and patients to understand that heart rate during exercise may not fully reflect exercise intensity in patients on beta-adrenergic blockers.

Retention rate over 80%, compliance rate of 100% to face-to-face exercise education and phone counseling sessions, and 83.1% of adherence rate in meeting exercise goals clearly showed that a home-based CR, implemented in this study was feasible. Knowing that only 61.5% participants were engaged in moderate-to vigorous intensity aerobic exercise for more than 150 min per week, and prior to the intervention, only 23.1% participants participated in both moderate- and vigorous intensity aerobic exercise for more than 150 min per week and resistance exercise for more than twice a week, at the end of the Study, 76.9% of participants met both aerobic and resistance exercise goals demonstrated that our home-based CR was not only feasible but also may be effective, although statistical analysis was not done due to the nature of the feasibility study. Furthermore, our survey results showed that participants in Study 2 were highly satisfied with the exercise education and telephone counseling provided. This suggests that a home-based exercise intervention supplemented with face-to-face exercise education sessions as well as exercise diary may be used for substitute for supervised exercise, although we did not compare its efficacy with supervised exercise group.

Most guidelines for CR exercise programs recommend a combination of aerobic and resistance exercises. The target intensity for aerobic exercises is usually set between 40 and 85% of either max heart rate or heart rate reserve [24]. The WHO recommends the highest intensity of exercise, expressed as 70–85% of heart rate peak, while Japan’s guidelines suggest the lowest intensity, expressed as 40–60% of heart rate reserve. However, these guidelines do not provide specific modifications for individuals using beta-adrenergic blockers, which can affect the recommended exercise intensity. Along with the guidelines for aerobic exercise, most countries, except Japan, have presented resistance exercise guidelines. The United States and the WHO have suggested calisthenic exercises either as the main exercise or as an additional activity [24]. Recently, calisthenic exercises have gained popularity among the general population in the era of the coronavirus pandemic. While calisthenic exercises have recently gained popularity, they have also been used as CR exercises [25–27] and present great potential for home-based CR programs. Calisthenic exercises do not require any equipment, making them accessible to anyone at home. If these calisthenic movements are strategically organized with varying repetitions and rest intervals [27], they can provide sufficient impact on the cardiopulmonary system, which is necessary for a proper CR program. Previously, we implemented a home-based exercise program consisting of calisthenic exercises supplemented with daily unsupervised walking exercises in patients with stage 1–3 colorectal cancer. Our study demonstrated that participants’ physical function significantly improved along with improvements in insulin resistance and adipocytokine levels [28, 29]. Taken together, the home-based exercise program used in this study was feasible and effective for increasing both aerobic and resistance exercises, which may lead to positive physiological changes in patients undergoing CR.

Our study has several limitations. First, this was a feasibility study conducted for 25 patients at a single tertiary hospital. Therefore, the results need to be confirmed in more patients in other institutes. Second, there was no control group; therefore, we cannot rule out the possibility that other factors such as weather and season may have influenced the results.

## Conclusions

In conclusion, we have designed and effectively implemented a home-based CR exercise program that proves to be feasible for patients with cardiovascular CVDs eligible for CR. Considering the insights gained from this study, it is recommended that future research endeavors focus on evaluating the effectiveness of home-based CR in influencing clinical outcomes.

### Supplementary Information

**Additional file 1: Supplementary Material l.** Aerobic exercise video. **Supplementary Material 2.** Resistance exercise. **Supplementary Material 3.** The TIDieR Checklist. **Supplementary Table 1.** Consensus of experts on the home-based cardiac rehabilitation (CR) program. **Supplementary Table 2.** Questionnaires regarding home-based aerobic and resistance exercise sessions (Study 1). **Supplementary Table 3.** Questionnaires regarding home-based aerobic and resistance exercise sessions (Study 2).

## Data Availability

The datasets used and/or analyzed during the current study are available from the corresponding author on reasonable request.

## Abbreviations

CR: Cardiac rehabilitation
MI: Myocardial infarction
CVD: Cardiovascular disease
AACVPR/AHA/ACC: American association of cardiovascular and pulmonary rehabilitation/american heart association/american college of cardiology
PA: Physical activity
WHO: World health organization
RPE: Rate of perceived exertion
HFrEF: Heart failure with reduced ejection fraction
GPAQ: Global physical activity questionnaire
