# Supplementary material for: Development and application of a home-based exercise program for patients with cardiovascular disease: a feasibility study
Source: BMC Sports Sci Med Rehabil. 2024 Feb 21;16:51. doi: 10.1186/s13102-024-00835-3 (PMC10880299; doi:10.1186/s13102-024-00835-3)
Supplement: Supplementary file 1 — Additional file 1: Supplementary Material l. Aerobic exercise video. Supplementary Material 2. Resistance exercise. Supplementary Material 3. The TIDieR Checklist. Supplementary Table 1. Consensus of experts on the home-based cardiac rehabilitation (CR) program. Supplementary Table 2. Questionnaires regarding home-based aerobic and resistance exercise sessions (Study 1). Supplementary Table 3. Questionnaires regarding home-based aerobic and resistance exercise sessions (Study 2). [file 13102_2024_835_MOESM1_ESM.docx]

Supplementary Material l. Aerobic exercise video


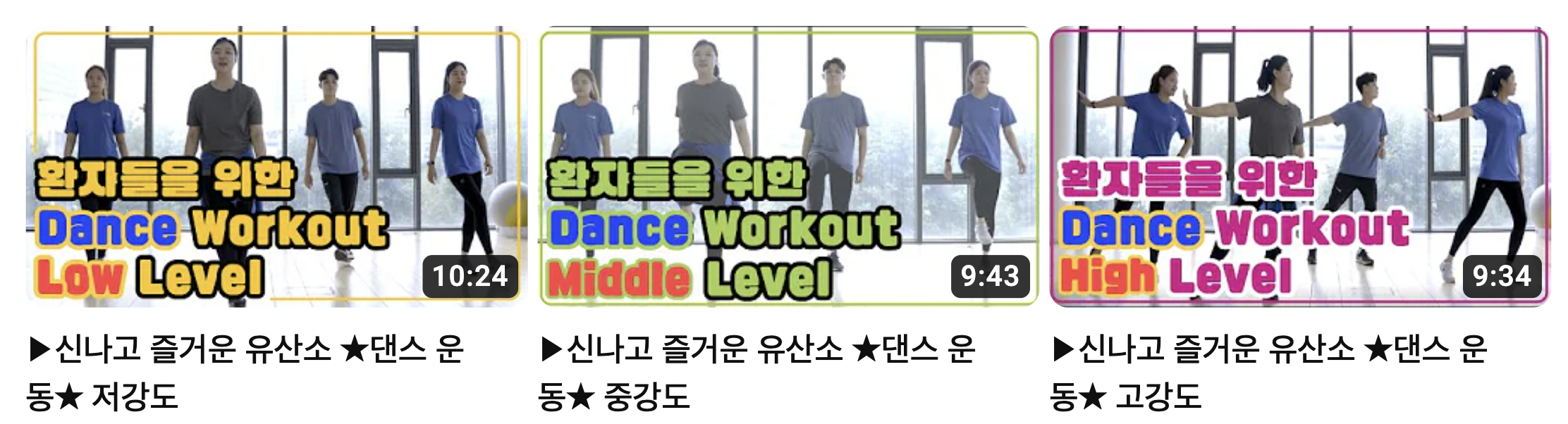


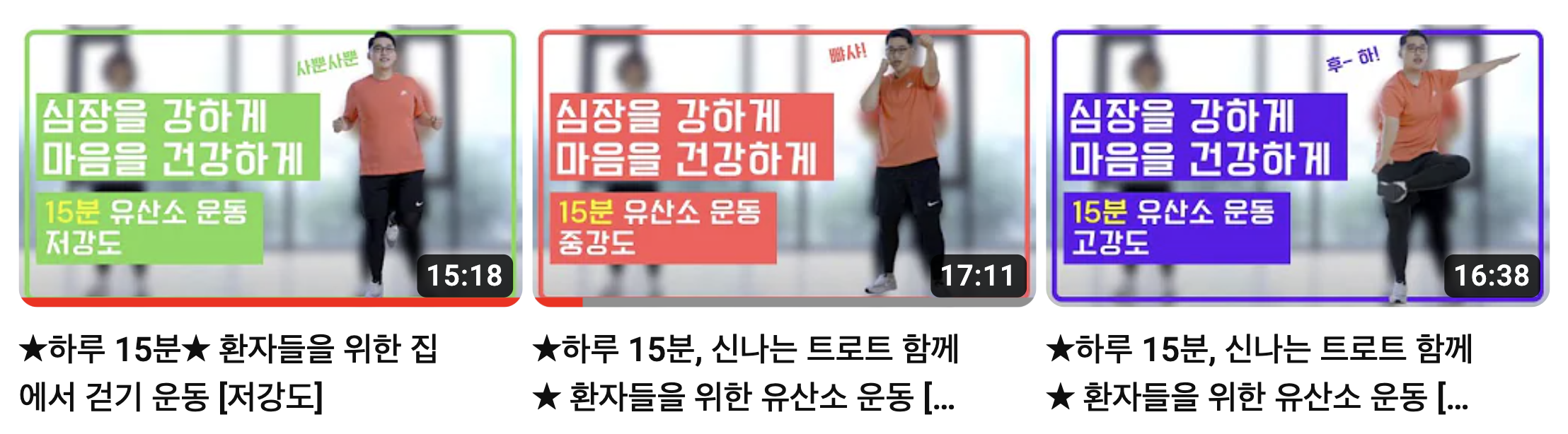


| 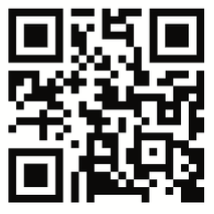 | 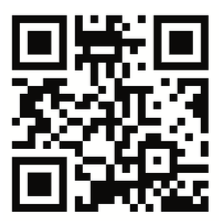 | 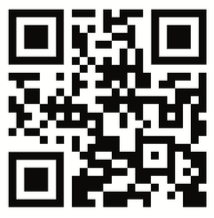 |
| --- | --- | --- |
| Low-intensity  aerobic exercise | Moderate-intensity  aerobic exercise | High-intensity  aerobic exercise |

Supplementary Material 2. Resistance exercise.
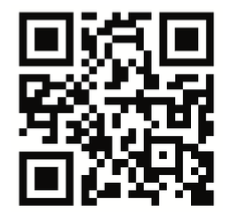

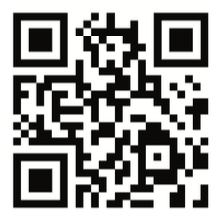


| 1. Child’s Pose and Cobra | 1. Pointer |
| --- | --- |
| 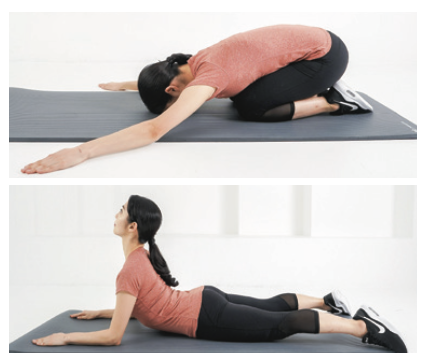 | 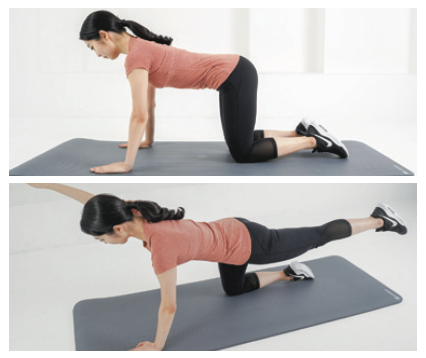 |
| 3.Push up | 4. Pelvic tilt |
| 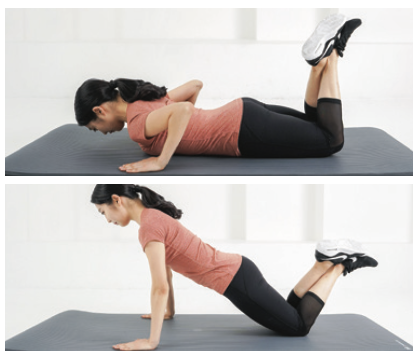 | 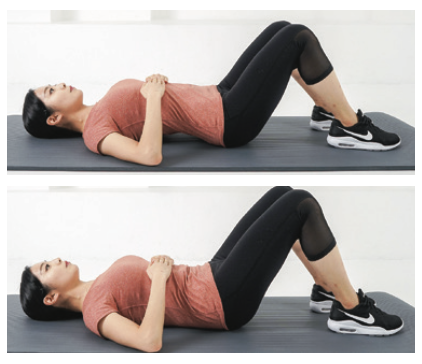 |
| 5. Curl up | 6. Bridge |
| 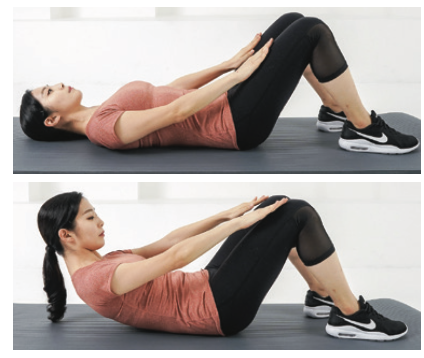 | 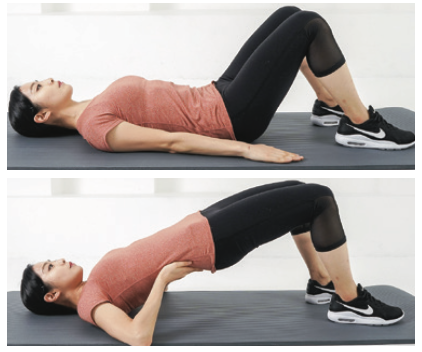 |
| 7. Squat | 8. Wall shoulder Press |
| 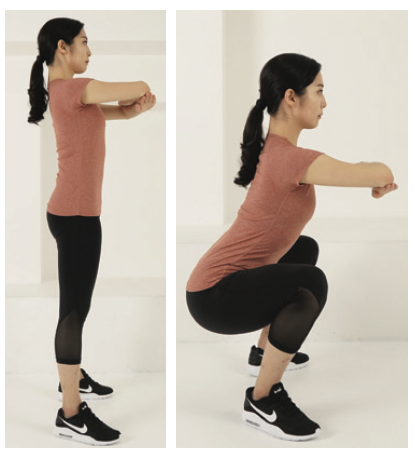 | 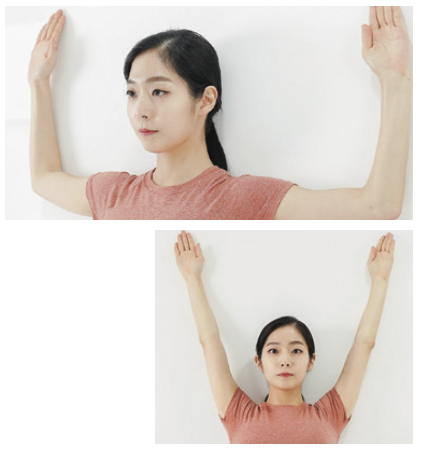 |

Supplementary Material 3. The TIDieR Checklist


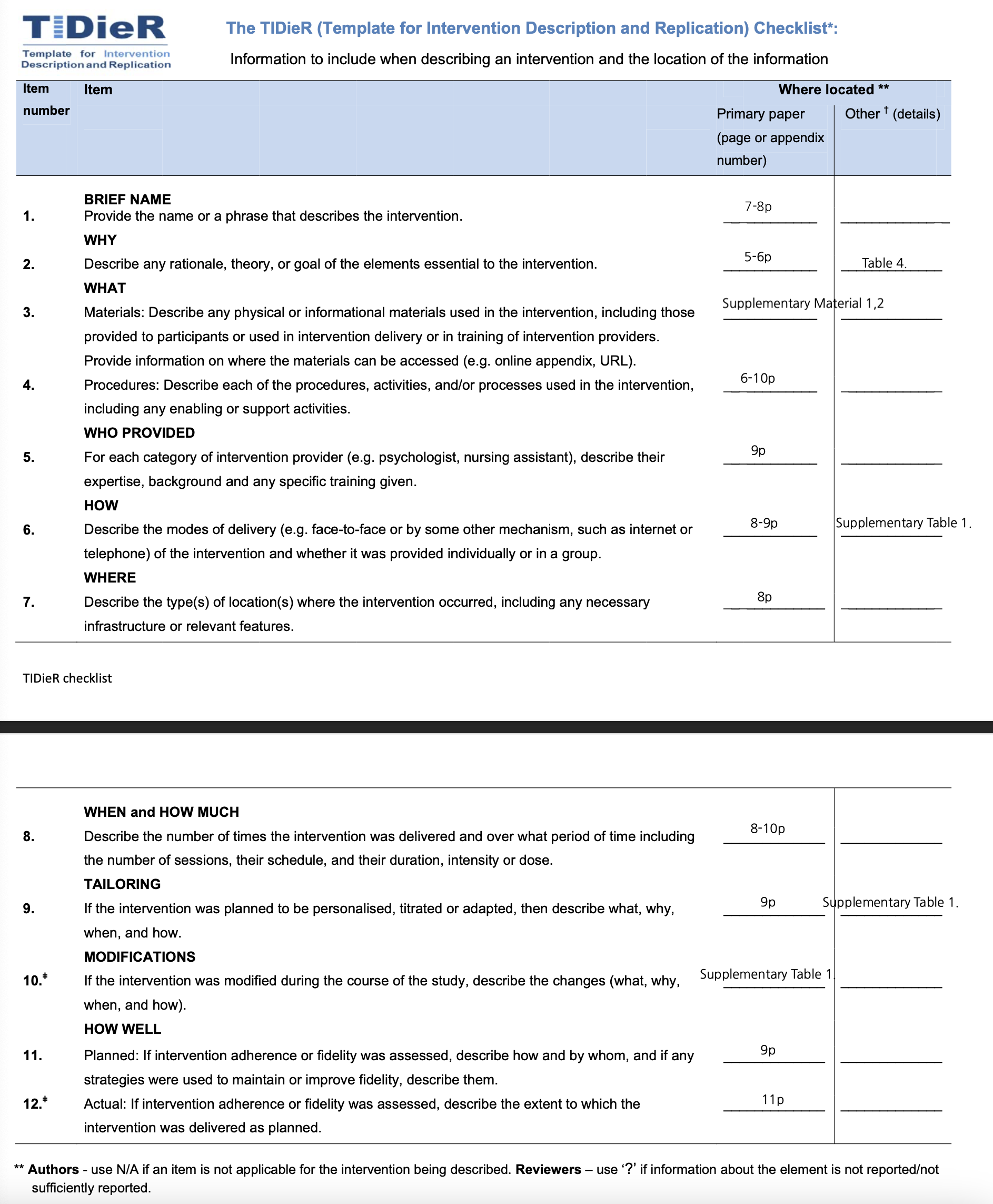


Supplementary Table 1. Consensus of experts on the home-based cardiac rehabilitation (CR) program

| Target home-based exercise goals | Rational |
| --- | --- |
| - 30-60 min walking per day up to 5 days per week, which could be divided up to 2-3 bouts depends on patients' fitness levels. - 1-2 sets calisthenics, composed of 8 exercises with rest interval (duration of rest interval is not set) | - 30-60 min up to 5 days per week, coincide with 150 min to 300 min of physical activity, physical activity guidelines by WHO and other relevant organization, such as American Heart Association, European Association of Cardiovascular Prevention and Rehabilitation and Canadian Association of CR) - Patients may break up their walking into 2-3 bouts, 10-15 min for each bout, which may increase the safety of program. - Use of calisthenic enable patients to participate in resistance exercise without any restrictions of equipment or places. |
| Consideration 1. Exercise environment: 1) Is there a treadmill or stationary bike at home? 2) Is outdoor walking feasible for patients? 3) Is there anyone patients can walk with? 4) Could patients have Smart TV, so that they can watch exercise video as they exercise. | |
| Consideration 2. Usage of exercise video  1) Exercise videos which participants can follow should be provided. There should be many tailored exercises videos which patients can follow including aerobic type and resistance type exercise. 2) Exercise video should include proper walking instruction. | |
| Consideration 3. Usage of heart rate monitor to determine exercise intensity.  1) Heart rate monitor should be provided, 2) Education session should include monitoring heart rate during walking at certain speed, 3) beta-blocker user should be instructed to use rate of perceived exertion and not solely depend on heart rate to determine exercise intensity | |
| Consideration 4. Joint conditions  1) Joint condition should be monitored, 2) Patients should be instructed to monitor their joint pain after exercise, 3) If joint pain worsen one day after walking, patients should be instructed to abstain from walking  4) If joint problem persist, patients should be encouraged to use stationary bike, elliptical or swimming | |
| Consideration 5. Identification of high-risk patients based on 6 min walk test (MWT) and left ventricular ejection fraction (LVEF)  1) No aerobic exercise if patient is able to walk less than 200 m during 6 MWT and LVEF less than 30%  2) Very Low intensity aerobic exercise if patient is able to walk between 200-300 m during 6 MWT and LVEF less than 30 %  3) Low intensity aerobic exercise if patient is able to walk more than 300 m during 6 MWT and LVEF less than 30 %  4) Very Low intensity aerobic exercise if patient is able to walk less than 200 m during 6 MWT and LVEF between 30-35%  etc. | |
| Consideration 6. Home-based CR exercise program should include followings:  1. Structure exercise program (FITT), 2. Lifestyle modification, 3. Awareness of adverse effects and their signs, 4. Patients' exercise preferences, 5. Accessibility to exercise facilities and exercise experts | |
| Consideration 7. Strategies should be developed and applied to increase compliance and adherence to exercise.   - Hybrid approaches: Supervise exercise education sessions and home-based exercise. - Usage of exercise diary and regular checking of the exercise diary - Regular exercise counselling over the phone (about 10 min, checking exercise compliances - Increase self-confidence and efficacy toward completing exercise program by providing tailored achievable exercise goals | |

Supplementary Table 2. Questionnaires regarding home-based aerobic and resistance exercise sessions (Study 1)

1. *Not satisfied at all ②Not satisfied ③ Neutral ④Satisfied ⑤ Very satisfied*

| 1. Stretching Satisfaction | ① | ② | ③ | ④ | ⑤ |
| --- | --- | --- | --- | --- | --- |
| Interest | ① | ② | ③ | ④ | ⑤ |
| Perceived intensity | ① | ② | ③ | ④ | ⑤ |
| Any suggestions |  |  |  |  |  |
| 2. Walking Satisfaction | ① | ② | ③ | ④ | ⑤ |
| Interest | ① | ② | ③ | ④ | ⑤ |
| Perceived Intensity | ① | ② | ③ | ④ | ⑤ |
| Any suggestions |  |  |  |  |  |
| 3. Aerobic Exercise Satisfaction | ① | ② | ③ | ④ | ⑤ |
| Interest | ① | ② | ③ | ④ | ⑤ |
| Perceived Intensity | ① | ② | ③ | ④ | ⑤ |
| Any suggestion |  |  |  |  |  |
| 4. Resistance Satisfaction | ① | ② | ③ | ④ | ⑤ |
| Interest | ① | ② | ③ | ④ | ⑤ |
| Perceived Intensity | ① | ② | ③ | ④ | ⑤ |
| Any suggestions |  |  |  |  |  |
| 5. General satisfaction of the exercise program | ① | ② | ③ | ④ | ⑤ |
| 6. Exercise video | ① | ② | ③ | ④ | ⑤ |
| 7. Perceived therapeutic benefit of exercise | ① | ② | ③ | ④ | ⑤ |
| 8. Willingness to exercise in the future | ① | ② | ③ | ④ | ⑤ |
| 9. Willingness to recommend this exercise to others | ① | ② | ③ | ④ | ⑤ |
| 10. Any other suggestions |  |  |  |  |  |

Supplementary Table 3. Questionnaires regarding home-based aerobic and resistance exercise sessions (Study 2)

1. *Not satisfied at all ②Not satisfied ③ Neutral ④Satisfied ⑤ Very satisfied*

| 1. Exercise Education Session Satisfaction | ① | ② | ③ | ④ | ⑤ |
| --- | --- | --- | --- | --- | --- |
| Duration | ① | ② | ③ | ④ | ⑤ |
| Any suggestion |  |  |  |  |  |
| 2. Telephone follow up Satisfaction | ① | ② | ③ | ④ | ⑤ |
| Frequency | ① | ② | ③ | ④ | ⑤ |
| Any suggestion |  |  |  |  |  |
| 4. Exercise diary Usefulness | ① | ② | ③ | ④ | ⑤ |
| 5. Exercise Video Usefulness | ① | ② | ③ | ④ | ⑤ |
| Any suggestions |  |  |  |  |  |
| 6. Any suggestion or comments on the home-based CR program | | | | | |
